# Deep Eutectic Solvents or Eutectic Mixtures? Characterization of Tetrabutylammonium Bromide and Nonanoic Acid Mixtures

**DOI:** 10.1021/acs.jpcb.2c00858

**Published:** 2022-05-24

**Authors:** Andrey Shishov, Patrycja Makoś-Chełstowska, Andrey Bulatov, Vasil Andruch

**Affiliations:** † Institute of Chemistry, Saint Petersburg State University, RU-198504 Saint Petersburg, Russia; ‡ Department of Process Engineering and Chemical Technology, Faculty of Chemistry, Gdańsk University of Technology, 80-233 Gdańsk, Poland; § EcoTech Center, Research Centre, Gdańsk University of Technology, G. Narutowicza St. 11/12, 80-233 Gdańsk, Poland; ∥ Department of Analytical Chemistry, Institute of Chemistry, Faculty of Science, Pavol Jozef Šafárik University in Košice, SK-04154 Košice, Slovak Republic

## Abstract

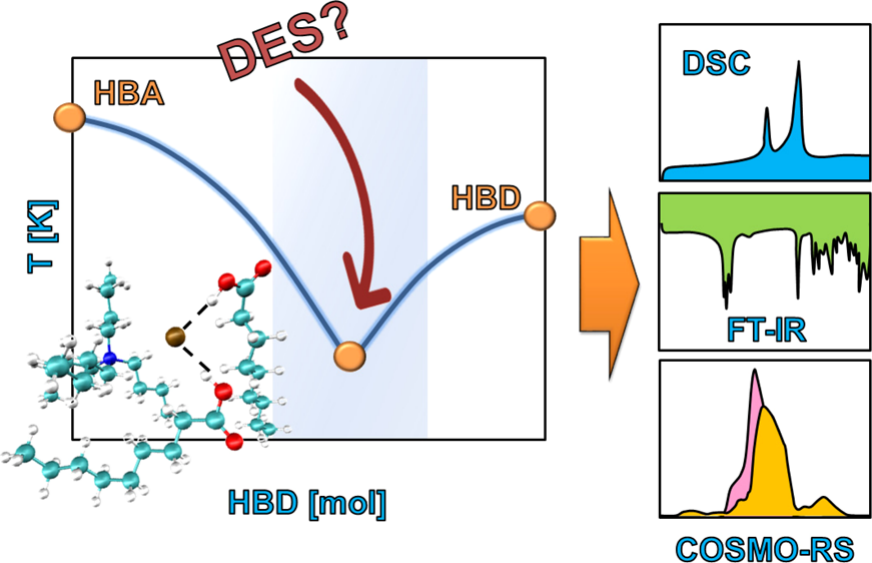

Deep eutectic solvents have quickly attracted the attention of researchers because they better meet the requirements of green chemistry and thus have the potential to replace conventional hazardous organic solvents in some areas. To better understand the nature of these mixtures, as well as expand the possibilities of their use in different industries, a detailed examination of their physical properties, such as density, viscosity, the nature of the interactions between their constituents, the phase diagrams, depression of their melting point, and interpretation of these results is necessary. In this work, the mixtures of tetrabutylammonium bromide (TBAB) and nonanoic acid (NA) in different molar ratios are theoretically and experimentally investigated by applying a phase diagram constructed on the basis of differential scanning calorimetry measurements and COSMO-RS model. Spectral properties are investigated based on Fourier transform infrared spectroscopy and density functional theory. The observed eutectic point indicates the formation of a DES in the TBAB−NA system in a 1:2 molar ratio. This is due to the presence of hydrogen bonds between the carboxyl group from the NA molecule and the bromine atom from the TBAB molecule. Other eutectic mixtures are most likely the solutions of TBAB in NA, in which hydrogen bonds predominate between acid molecules.

## 1 Introduction

Organic solvents are widely used in various industries as well as in research. At present, society has placed great emphasis on sustainable development.^1^ Therefore, lately, we can observe a clear effort to replace hazardous solvents with environmentally friendly ones that meet the requirements of green chemistry.^2^ From this point of view, the so-called deep eutectic solvents (DESs), which are characterized by some interesting properties, can be considered among the promising groups of solvents. Probably, the most interesting feature of DESs is their tunability, that is, the ability to change the properties of a solvent by the choice of the hydrogen bond acceptor (HBA) and the hydrogen bond donor (HBD) and their molar ratios. DESs were designed by the Abbott’s group in the beginning of the century.^3,4^ One of the first DESs studied was composed of choline chloride and urea,^4^ and choline chloride-based DESs probably remain the most studied DESs to date. Since then, many different DESs have been designed and researched.^5,6^

DESs, like many other ideas in history, have begun to be researched, developed, and used through a variety of applications. However, for further progress, it is necessary to study basic issues, such as the origin of a DES, the nature of the interactions between their components, their supramolecular structure, phase diagrams, and interpretation of these results.^7−9^ The DESs consist of a mixture of two or more solid components that give rise to a lower melting point compared to the starting materials^10^ for which the eutectic point temperature is lower than the temperature of an ideal liquid mixture.^11^ In order to correctly assess which mixture is really DES, a series of tests on new combinations should be performed each time. Theoretically, the preparation of a phase diagram is the best tool for assessment.^5,12^

DESs based on various quaternary ammonium salts are quite often used. The mixtures of tetrabutylammonium bromide and ethylene glycol,^13−18^ 1,3-propanediol,^13,14,18^ 1,5-pentanediol,^13,14,18^ glycerol,^13−16,18−20^ imidazole,^15,21^ triethylene glycol,^22^ carboxylic acid,^23^ and polyethylene glycol^24,25^ were investigated. Therefore, in this work, we investigate mixtures of tetrabutylammonium bromide (TBAB) and nonanoic acid (NA) in various molar ratios using a phase diagram constructed by means of differential scanning calorimetry (DSC) thermograms as well as spectral properties based on Fourier-transform infrared spectroscopy (FT-IR). Experimental results were compared with theoretical calculations. To the best of our knowledge, the TBAB–NA system has not yet been studied from this point of view.

## 2 Experimental Section

### 2.1 Chemicals

TBAB (98%; Sigma-Aldrich, Germany) and NA (99.5%; Sigma-Aldrich, Germany) were used as supplied.

### 2.2 Preparation of DESs

The DESs were prepared by mixing the appropriate amounts of the two components at an elevated temperature. The required amounts of TBAB as the HBA and NA as the HBD in various molar ratios from 1:1 to 1:30 were introduced into laboratory glass vials. The first component (TBAB) was weighed directly into the glass vial. The second component (NA) was then added dropwise using an automatic pipette to the same vial placed on the analytical balance. Subsequently, a magnetic stirrer was inserted, and the vial was placed on a magnetic stirrer with heating. The mixture was stirred at 300 rpm and a temperature up to 80 °C using a temperature-controlled magnetic stirrer (model RHD, IKA, Germany) until a homogenous liquid was formed. The temperature increases, the viscosity of NA is decreased, and the solubility of TBAB is increased; as a result, the formation of the eutectic mixture proceeds faster. After the preparation of the DESs, they were cooled to room temperature, closed with polypropylene caps, and kept closed at room temperature.

### 2.3 Apparatus

DSC experiments were carried out using a Netzsch DSC 204 F1 Phoenix calorimeter. The samples were investigated in 40 μL of aluminum crucibles with pierced lids. The rate of temperature change was 5 K·min^−1^ in the temperature region 203 to 332 K (218 to 405 K for TBAB). The behavior of eutectic solvents at higher rates (10 K·min^−1^) and lower rates (1 K·min^−1^) has been studied. At the same time, no significant change in the position and magnitude of the peaks was observed. All samples were first cooled and then heated. A total of one cooling and heating cycle was performed. The presented data were obtained on a heating cycle.

FT-IR spectra of the DESs as well as pure NA were recorded using a Bruker Tensor 27 spectrometer (Bruker, USA) with an ATR accessory and OPUS software (Bruker, USA). The following operating parameters were used: spectral range 4000–550 cm^–1^, resolution: 4 cm^–1^, number of sample scans: 256, number of background scans: 256, and slit width: 0.5 cm. An 831KF Coulometer (Metrohm, Switzerland) was used for the determination of water in DESs.

### 2.4 Theoretical FT-IR Bands

Theoretical FT-IR vibrational bands calculation of the DESs were performed according to a previous work.^19^ In the first step, the structures of all DESs were generated by the Avogadro 1.2.0 software.^26^ The molecular structures of the DESs were geometry-optimized using B3LYP/6-31+G** with a dispersion-corrected computational model using the Orca 4.1.1 program.^27,28^ All configurations were tested to be local minima by frequency calculations. The atomic displacements for the vibrational DESs mode were calculated using Multiwfn 3.7 software, and two factors, 0.958 and 0.983, were used to scale the frequencies.^29,30^

### 2.5 Phase Diagram Calculation

The theoretical phase diagram of TBAB:NA was prepared using a conductor-like screening model for real solvents (COSMO-RS) based on previous studies.^31,32^ Each calculation was prepared using the ADF COSMO-RS software (SCM, Netherlands). DES conformers were generated using the BP-TZVP level of theory. The molecules were modeled by COSMO-RS using the individual ions approach. Each of the ions was optimized separately, and the salt was treated as a mixture of two ions with an appropriate molar ratio, according to the previous study.^33^ The sigma profile surfaces are presented in Figure S1. In this approach, ions are treated at the quantum chemical level separately, which allows studying the contribution of both the cation and anion to predict the interaction in the DES mixture. The plot of the phase diagram was prepared based on eq 1, in which the change in calorific capacity (Δ*C*_*p*_) was neglected due to the fact that this parameter is not readily available in the literature and that it is difficult to measure.^32,34−37^
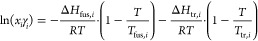
1where *x_i_* is the molar ratio of component *i*, γ_*i*_ is the activity coefficient of component *i*, Δ*H*_fus,*i*_ is the enthalpy of fusion of component *i* [J·mol^–1^], Δ*H*_tr,*i*_ is the solid–solid transition enthalpy of component *i* [J·mol^–1^], *R* is the universal gas constant [8.314 J·mol^–1^·K^–1^], *T* is the temperature of the system [K], *T*_fus,*i*_ is the melting point temperature of component *i* [K], and *T*_tr,*i*_ is the solid–solid transition temperature of component *i* [K].

## 3 Results and Discussion

### 3.1 Water Content, Density, and Viscosity Measurements

The physicochemical properties of DESs can be influenced by the water content.^15,38^ Therefore, the water content was measured in starting substances and prepared DESs immediately after the preparation using the Karl Fischer titration method (Table 1).

**Table 1:** Water Content, Density and Viscosity of the Studied DESs and Starting Materials

DES (molar ratio)	water content (ppm)	density at 40 °C (g·cm^–3^)	viscosity at 40 °C (mPa·s)
TBAB	4115		
TBAB−NA (1:1)	2229	0.9891	124.04
TBAB−NA (1:2)	1724	0.9709	34.03
TBAB−NA (1:3)	1619	0.9491	14.05
TBAB−NA (1:4)	1369	0.9265	10.01
TBAB−NA (1:5)	1216	0.9178	8.24
TBAB−NA (1:10)	829	0.9044	6.16
TBAB−NA (1:15)	806	0.8982	5.75
TBAB−NA (1:20)	669	0.8965	5.61
TBAB−NA (1:25)	641	0.8923	5.36
TBAB−NA (1:30)	633	0.8911	5.33
NA	628	0.8905	4.72

**Figure 1:**
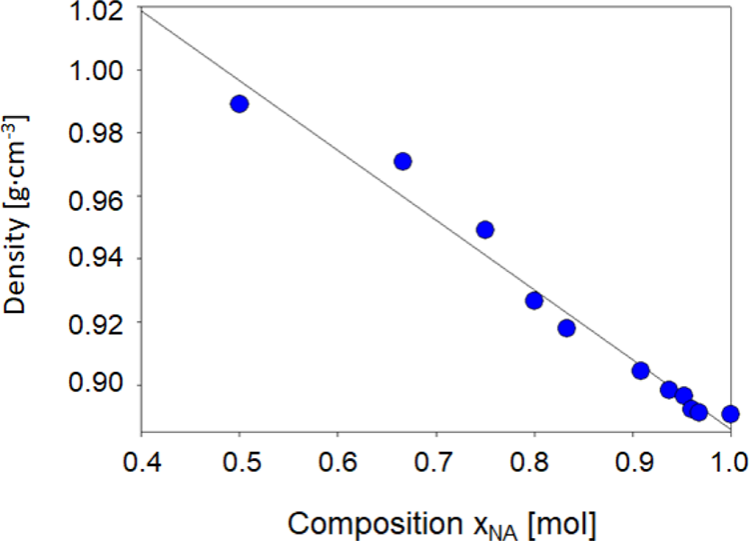
Density of the studied DESs.

**Figure 2:**
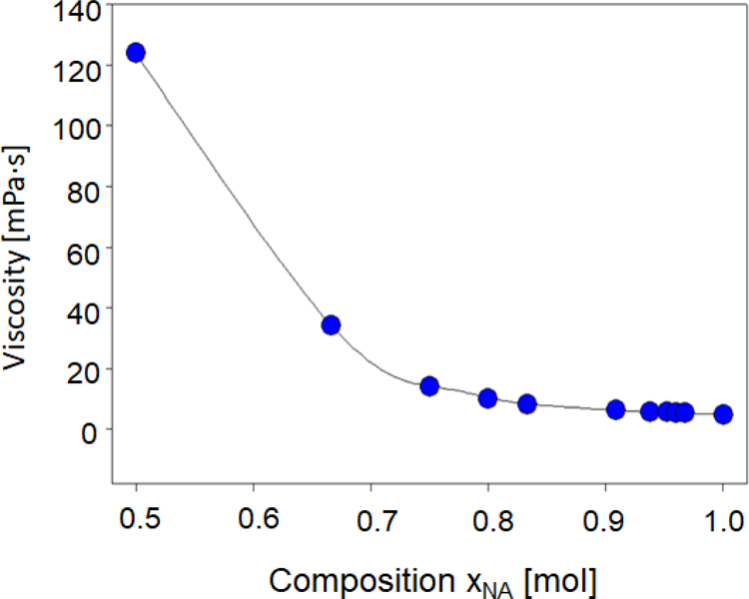
Viscosity of the studied DESs.

The densities decrease practically linearly with increasing mole fraction of NA (Figure 1). This is probably due to the fact that as the amount of NA in the DES structure increases, the available free volume of DES increases. The viscosity decrease (Figure 2) is much faster than the density decrease. The decrease in viscosity along with the increase in the NA content in the DES is probably due to the increase in the strength of the intermolecular interactions between TBAB–NA and NA–NA.

### 3.2 Structural Characterization

**Figure 3:**
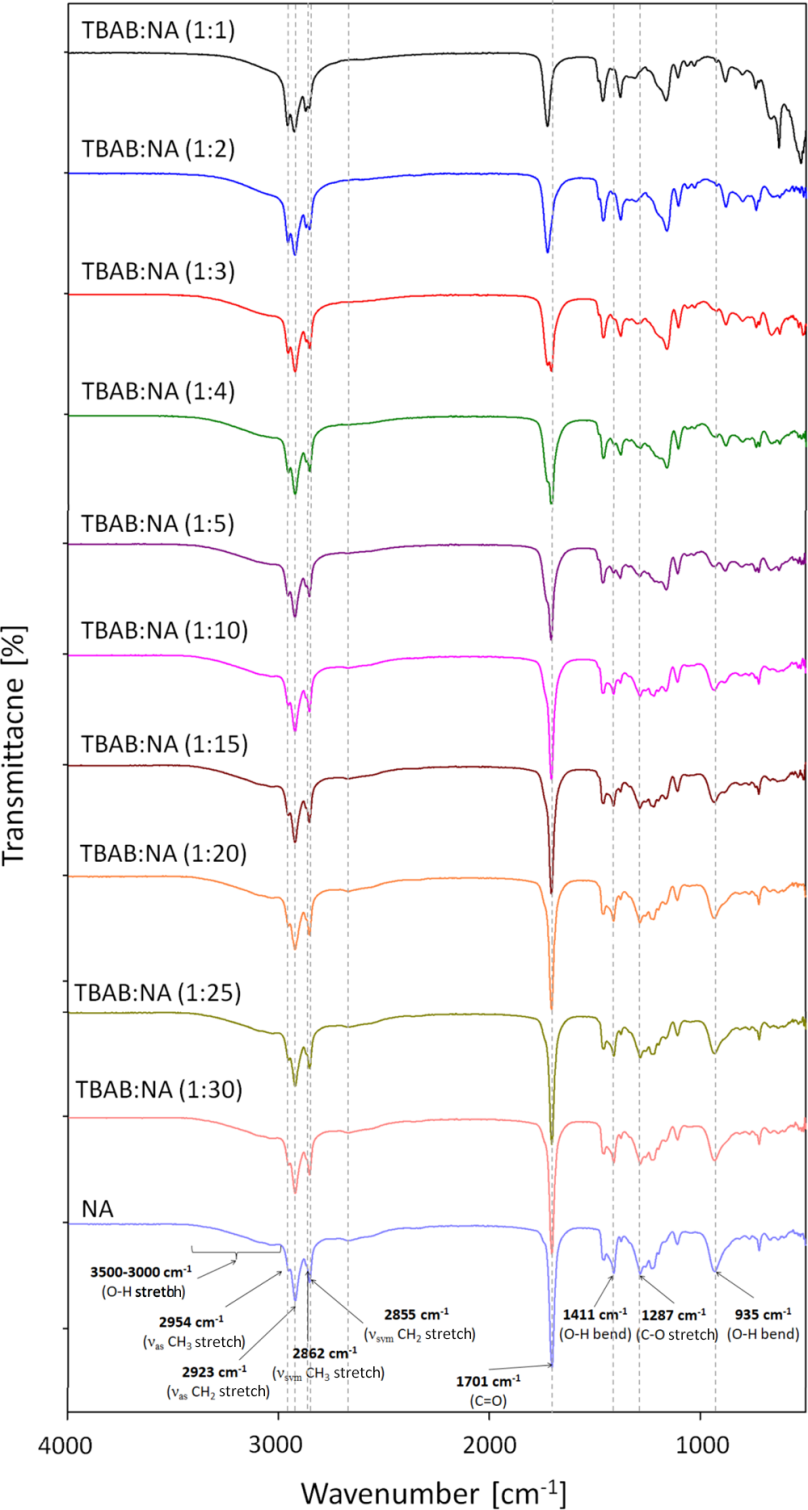
FT-IR spectra of DESs composed of TBAB and NA in various molar ratios and pure NA.

The chemical structures of DESs based on TBAB and NA in various molar ratios were confirmed by FT-IR studies. All obtained spectra are presented in Figure 3. The list of FT-IR frequencies is given in Table 2. In the spectra of pure NA (light blue), the characteristic C–H stretching vibrations (asymmetric CH_3_, CH_2_, and symmetric CH_3_, CH_2_) are observed in the region of 2954–2855 cm^–1^. However, after the addition of TBAB to NA, the position of these peaks do not change, and they overlap with C–H stretching vibrations from pure TBAB^19^. This indicates that hydrocarbon groups (C–H) from TBAB and NA are not responsible for DES formation. In all spectra, a wide peak in the region of 3500–3000 cm^–1^ is observed. In the pure NA spectrum, this peak can be attributed to the O–H stretch, which is characteristic of carboxylic acids.^39^ In the TBAB−NA spectra, the peak of the O–H stretch overlaps with the N–H stretch from TBAB.^19^ However, in both the pure NA and TBAB spectra, peaks in the region of 3000–3500 cm^–1^ are very broad, and it is very difficult to identify their characteristic wavelength. Therefore, the other bands should be considered to determine the interactions between TBAB and NA in DES structures. One of the characteristic peaks with high intensity in the NA spectrum is observed at 1701 cm^–1^ and can be attributed to C=O stretching. In the spectra of TBAB−NA mixtures with TBAB:NA molar ratios of 1:30, 1:25, 1:20, 1:15, and 1:10, this peak remains in the same position. In turn, in the spectra of TBAB−NA with TBAB:NA molar ratios of 1:5, 1:4, 1:3, 1:2, and 1:1, the peak is shifted toward higher wavenumber values ranging from 1701 to 1728 cm^–1^. The opposite behavior was obtained for the C–O stretch and O–H bends, which can be observed at 1287 cm^–1^ (νC–O), 1411 cm^–1^ (δO–H) and 935 cm^–1^ (δO–H), respectively, in the spectrum of pure NA. In the spectra of a DES composed of TBAB−NA with TBAB:NA molar ratios in the range of 1:1–1:5, the peaks of the νC–O, δO–H and δO–H groups are shifted toward lower wavenumber values. Further increasing the amount of TBAB in the DES complex does not change the position of the peak. The obtained results indicate the gradual distribution of the −COOH group, which is caused by the H-bond in the DES structure formation. Based on the obtained spectra and the previous studies, it can be concluded that a hydrogen bond in DES complexes is formed between the carboxyl group from NA molecule and the bromine atom from TBAB molecule.^40^ The bromine atom is not visible in the FT-IR spectrum. If a hydrogen bond formed between the −COOH and other TBAB groups, it would shift the visible bands in the FT-IR spectra. In addition, only in DESs composed of TBAB−NA with TBAB:NA molar ratios of 1:1, 1:2, 1:3, 1:4, and 1:5, can it be concluded that hydrogen bonds are formed between the HBA and the HBD. The formation of hydrogen bonds is a necessary condition for the formation of the DES. In TBAB−NA with TBAB:NA molar ratios of 1:10 to 1:30, the spectra practically do not differ from the pure NA spectrum. Therefore, it is probable that the remaining TBAB−NA complexes in a 1:10 to 1:30 molar ratio are most likely solutions of TBAB in NA, in which hydrogen bonds between the acid molecules predominate.

**Table 2:** Experimental and Calculated FT-IR Frequencies [cm^–1^] for the Main Groups of Pure NA, Pure TBAB,^19^ and TBAB−NA DESs with Various TBAB:NA Molar Ratios (The Calculated Values after Scaling Are Given in Parentheses)

mode	NA	TBAB^19^	1:1	1:2	1:3	1:4	1:5	1:10	1:15	1:20	1:25	1:30
N−H/O–H stretch	3300–3500^a^	3415^b^	3300–3500^c^	3300–3500^c^	3300–3500^c^	3300–3500^c^	3300–3500^c^	3300–3500^c^	3300–3500^c^	3300–3500^c^	3300–3500^c^	3300–3500^c^
	*(3300–3500)^a^*	*(3419)^b^*	*(3300–3500)*^c^	*(3300–3500)*^c^	*(3300–3500)*^c^	*(3300–3500)*^c^	*(3300–3500)*^c^	*(3300–3500)*^c^	*(3300–3500)*^c^	*(3300–3500)*^c^	*(3300–3500)*^c^	*(3300–3500)*^c^
asymmetric CH_3_ stretch	2954	2955	2955	2955	2955	2955	2955	2954	2954	2954	2954	2954
	*(2956)*	*(2957)*	*(2956)*	*(2956)*	*(2956)*	*(2956)*	*(2956)*	*(2956)*	*(2956)*	*(2956)*	*(2956)*	*(2956)*
asymmetric CH_2_ stretch	2923	2935	2923	2923	2923	2923	2923	2923	2923	2923	2923	2923
	*(2926)*	*(2936)*	*(2925)*	*(2925)*	*(2925)*	*(2925)*	*(2925)*	*(2926)*	*(2926)*	*(2926)*	*(2926)*	*(2926)*
symmetric CH_3_ stretch	2862	2880	2862	2862	2862	2862	2862	2862	2862	2862	2862	2862
	*(2863)*	*(2881)*	*(2864)*	*(2864)*	*(2864)*	*(2864)*	*(2864)*	*(2863)*	*(2863)*	*(2863)*	*(2863)*	*(2863)*
symmetric CH_2_ stretch	2855	2872	2855	2855	2855	2855	2855	2855	2855	2855	2855	2855
	*(2858)*	*(2873)*	*(2857)*	*(2857)*	*(2857)*	*(2857)*	*(2857)*	*(2858)*	*(2858)*	*(2858)*	*(2858)*	*(2858)*
C=O stretch	1701		1728	1728	1728	1709	1709	1707	1707	1707	1706	1701
	*(1705)*		*(1729)*	*(1729)*	*(1727)*	*(1711)*	*(1710)*	*(1709)*	*(1709)*	*(1709)*	*(1707)*	*(1703)*
CH_3_ and CH_2_ scissors	1490–1462	1492–1445	1490–1445	1490–1445	1490–1445	1490–1445	1490–1445	1490–1446	1490–1462	1490–1462	1490–1462	1490–1462
	*(1491–1466)*	*(1492–1444)*	*(1490–1446)*	*(1490–1446)*	*(1490–1446)*	*(1490–1446)*	*(1490–1446)*	*(1490–1447)*	*(1490–1463)*	*(1490–1463)*	*(1490–1463)*	*(1490–1463)*
C–H bend	1425	1425	1425	1425	1425	1425	1425	1425	1425	1425	1425	1425
	*(1422)*	*(1426)*	*(1426)*	*(1426)*	*(1426)*	*(1426)*	*(1425)*	*(1422)*	*(1422)*	*(1422)*	*(1422)*	*(1422)*
C–OH stretch	1413		1381	1381	1388	1388	1388	1410	1410	1410	1411	1413
	*(1416)*		*(1383)*	*(1383)*	*(1389)*	*(1389)*	*(1389)*	*(1412)*	*(1412)*	*(1412)*	*(1413)*	*(1416)*
CH_2_ twist	1336	1335	1335	1335	1335	1335	1335	1336	1336	1336	1336	1336
	*(1335)*	*(1333)*	*(1333)*	*(1333)*	*(1333)*	*(1333)*	*(1333)*	*(1335)*	*(1335)*	*(1335)*	*(1335)*	*(1335)*
C–O stretch	1287		1263	1263	1263	1263	1282	1286	1287	1287	1287	1287
	*(1289)*		*(1266)*	*(1266)*	*(1266)*	*(1266)*	*(1284)*	*(1288)*	*(1289)*	*(1289)*	*(1289)*	*(1289)*
asymmetric and symmetric CH_3_ rock	1188–1075	1185–1071	1186–1072	1186–1072	1186–1072	1186–1073	1186–1074	1188–1075	1188–1075	1188–1075	1188–1075	1188–1075
	*(1190–1076)*	*(1191–1076)*	*(1188–1073)*	*(1188–1073)*	*(1188–1073)*	*(1188–1074)*	*(1188–1075)*	*(1190–1076)*	*(1190–1076)*	*(1190–1076)*	*(1190–1076)*	*(1190–1076)*
CH_2_ twist	1058	1056	1057	1057	1057	1057	1057	1058	1058	1058	1058	1058
	*(1061)*	*(1058)*	*(1059)*	*(1059)*	*(1059)*	*(1059)*	*(1059)*	*(1061)*	*(1061)*	*(1061)*	*(1061)*	*(1061)*
asymmetric C–N stretch		1021 *(1023)*	1021 *(1022)*	1021 *(1022)*	1021 *(1022)*	1021 *(1022)*	1021 *(1022)*					
CH_3_ rock	1006	1005	1005	1005	1005	1005	1005	1006	1006	1006	1006	1006
	*(1008)*	*(1007)*	*(1007)*	*(1007)*	*(1007)*	*(1007)*	*(1007)*	*(1008)*	*(1008)*	*(1008)*	*(1008)*	*(1008)*
CH_2_ rock	975–926	975–926	975–926	975–926	975–926	975–926	975–926	975–926	975–926	975–926	975–926	975–926
	*(973–929)*	*(973–929)*	*(973–929)*	*(973–929)*	*(973–929)*	*(973–929)*	*(973–929)*	*(970–930)*	*(970–930)*	*(970–930)*	*(970–930)*	*(970–930)*
O–H bend	933		888	889	889	889	900	935	935	935	935	935
	*(932)*		*(889)*	*(889)*	*(889)*	*(890)*	*(902)*	*(936)*	*(936)*	*(936)*	*(936)*	*(936)*
C–C stretch	899	898	898	898	898	898	898	899	899	899	899	899
	*(904)*	*(902)*	*(901)*	*(901)*	*(901)*	*(901)*	*(901)*	*(904)*	*(904)*	*(904)*	*(904)*	*(904)*
NC_4_ symmetric stretch		712	712	712	712	712	712					
		*(710)*	*(710)*	*(710)*	*(710)*	*(710)*	*(710)*					
CCC deformation	556	550	555	555	555	555	555	556	556	556	556	556
	*(558)*	*(558)*	*(557)*	*(557)*	*(557)*	*(557)*	*(557)*	*(558)*	*(558)*	*(558)*	*(558)*	*(558)*

a O–H stretch frequency for NA.

b N–H stretch frequency for TBAB.

c Overlapping N–H and O–H stretch frequencies.

The obtained theoretical results indicate that the position of the main bands is shifted toward higher wavenumbers compared to experimental studies. This is due to the fact that the theoretical calculations have been made for free TBAB−NA complexes in a vacuum, while experimental studies were performed for liquid DESs.^41,42^ Therefore, the calculated results were scaled by empirical scaling factors. The factor of 0.958 was adopted for the range of 4000 to 1700 cm^–1^ wavenumber, while a 0.983 factor was used for scaling wavenumbers from 1700 to 500 cm^–1^, based on the previous studies.^19,30^ A list of the experimental and calculated FT-IR frequencies for the main groups of pure NA, pure TBAB, and DESs composed of TBAB and NA in various molar ratios is presented in Table 2. The theoretical results differ by a maximum of 3 cm^–1^ compared to experimental FT-IR spectra.

**Figure 4:**
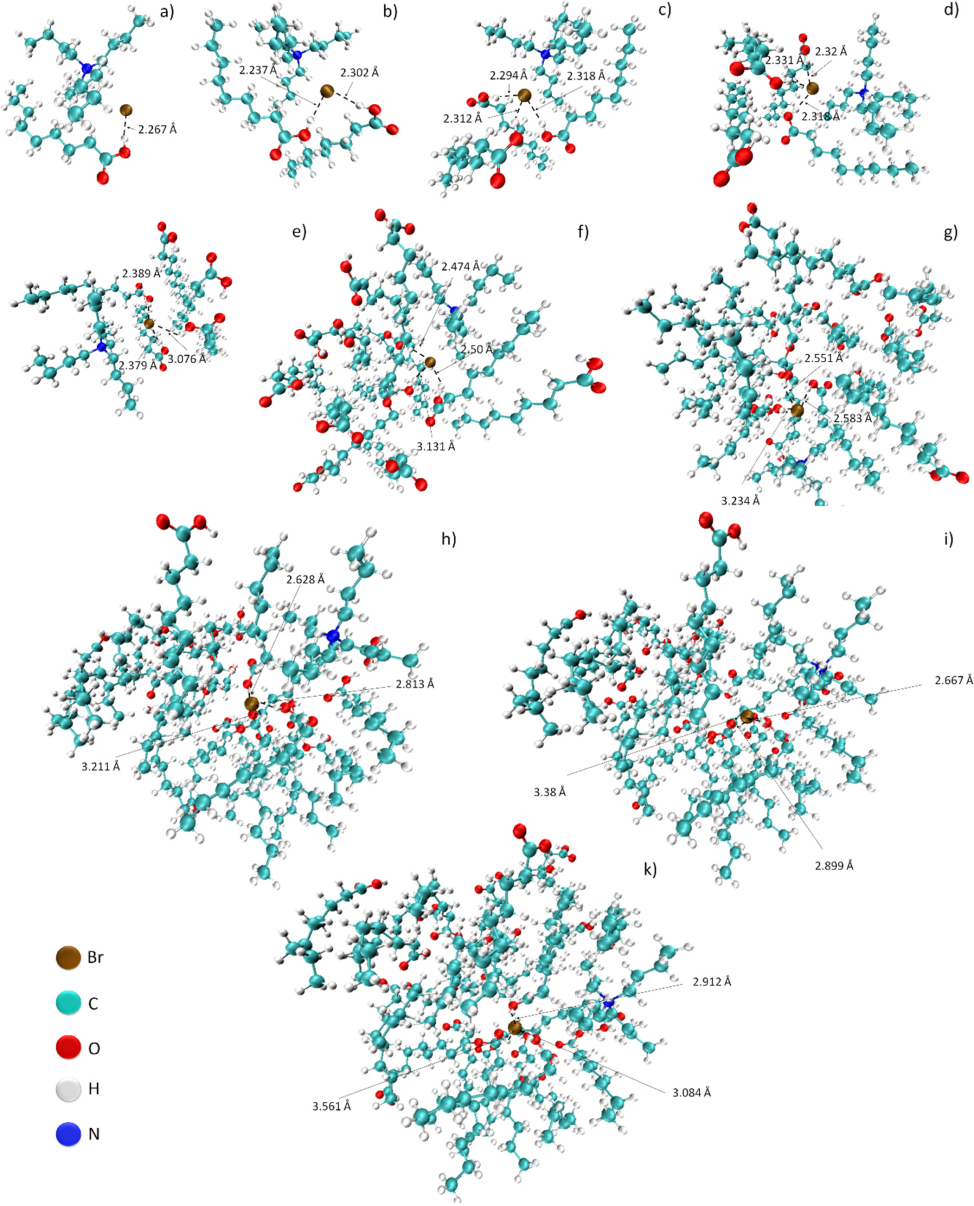
Structures of TBAB−NA complexes with TBAB:NA molar ratios of (a) 1:1, (b) 1:2, (c) 1:3, (d) 1:4, (e) 1:5, (f) 1:10, (g) 1:15, (h) 1:20, (i) 1:25, and (k) 1:30.

The modeled structures of the DES molecules are shown in Figure 4. It can be observed that H-bonds are formed between the Br atom and −COOH groups in TBAB−NA with 1:1, 1:2, 1:3, 1:4, and 1:5 molar ratios because of the short distance (below 2.5 Å). In TBAB−NA with 1:1, 1:2, and 1:3 molar ratios, each of the carboxylic acids formed H-bonds with the bromide atom in TBAB. A further increase in the number of acid molecules in DES structures causes gradual elongation of the existing hydrogen bonds. In complexes with TBAB:NA molar ratios of 1:10–1:30, the bond lengths between the Br^–^ atom and the −COOH group exceed 2.5 Å. This means that hydrogen bonds are transformed into weaker van der Waals interactions. However, we can observe the formation of new type of H-bond interaction between only carboxylic groups of NA. Interactions between carboxyl groups begin to dominate the molecule, creating solutions of TBAB in NA. Therefore, the theoretical results confirm the conclusions obtained in the experimental research.

### 3.3 Phase Diagram

The main property of DESs in comparison with other solvents and mixtures is a sharp decrease in the melting point of such a system in comparison with the initial substances. The best method for determining the melting point and other phase transitions is DSC.

**Figure 5:**
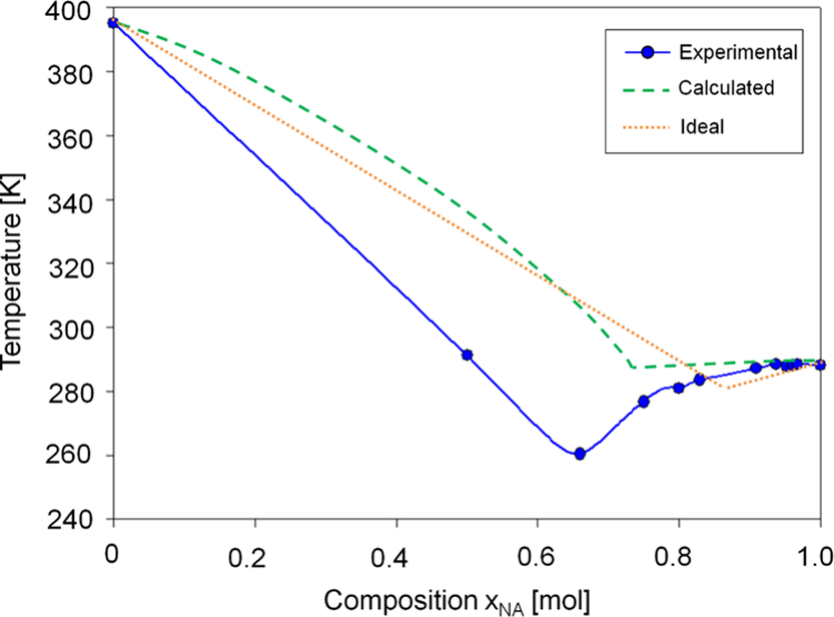
Experimental and calculated based COSMO-RS model phase diagram.

From the data obtained (See Electronic Supplementary, Figure S1), it can be concluded that tetrabutylammonium has two characteristic peaks, at 364 and 393 K, which corresponds to the crystal readjustment and conformational (first peak) and the melting of the amine itself (second peak).^43^ In the case of NA, there are two characteristic peaks, at 269 and 290 K. Based on previous studies, the first and the second peak show a lower and higher temperature than the typical nonanoic acid melting point, which is 285 K. This can be explained by the occurrence of a solid–solid phase change.^44^ In the case of eutectic mixtures in the range from 1:1 to 1:3, a new peak gradually appears at a temperature of around 260 K (Figure S1). With a further increase in the acid fraction, a second peak appears at a temperature of around 277 K, which is responsible for the phase transition of the free acid. With a further increase in the NA content of the complexes, the second peak shifts from 277 to 288 K. Thus, it can be concluded that at TBAB:NA molar ratios from 1:1 to 1:3, all of the nonanoic acid is used in the formation of the DES phase, and with its further increase, the DES phase dissolves in an excess of acid. The melting points of DESs in various molar ratios are plotted on the phase diagram (Figure 5). The results indicate that a DES based on TBAB−NA is characterized by large melting point depressions, which are characteristic of deep eutectic systems. These results can be explained by the cross-interaction between the quaternary ammonium salt (TBAB) and the carboxylic acid (NA).

In the next part of the study, theoretical solid–liquid phase diagrams were prepared using two methodologies based on eq 1, including (a) the assumption of an ideal liquid phase where γ_*i*_ = 1 and (b) calculation of the activity coefficients through the associative COSMO-RS. The values of the fusion (melting) point, solid–solid transition temperature, the enthalpy of fusion, and solid–solid transition enthalpy for pure TBAB were adopted as *T*_fus,*i*_ = 395 K, *T*_tr,*i*_ = 293 K, Δ*H*_fus,*i*_ = 16,150 J·mol^–1^, Δ*H*_tr,*i*_ = 67 J·mol^–1^, and for NA as *T*_fus,*i*_ = 285 K, *T*_tr,*i*_ = 283 K, and Δ*H*_fus,*i*_ = 20,310 J·mol^–1^, Δ*H*_tr,*i*_ = 20,290 J·mol^–1^.^45,46^ The calculations were prepared for DES containing 0.1, 0.2, 0.3, 0.4, 0.5, 0.6, 0.7, 0.8, and 0.9 mol of NA in DES complexes. Experimental, ideal, and theoretical phase diagrams are presented in Figure 5. The obtained results indicate that the depressions of the calculated, ideal, and experimental melting point slightly differ. In the experimental phase diagram, the lowest temperature depression can be observed for TBAB−NA with a TBAB:NA molar ratio of 1:2, while in the computational and ideal diagrams it occurs at molar ratios of 1:3 and 1:2.5, respectively. In addition, the difference in the value of the melting points at the high depression point can be observed. In the calculated and ideal diagrams, the eutectic points are 290 and 280 K. They are 30 and 20 K higher than in the DSC studies. However, the calculated values represent an ideal liquid mixture. The nonideality of the phase behavior of the real DES can be explained by the contributions of excess enthalpy and entropy.^12,47^ According to the definition of DES, there is a eutectic mixture of two or more components for which the eutectic point temperature must be lower than that of an ideal liquid mixture.^11^ These results indicate that it is indeed a deep eutectic mixture and not a simple eutectic mixture.

## 4 Conclusions

In this work, a mixture of TBAB and NA in different molar ratios in the range from 1:1 to 1:30 was studied using DSC measurements, FT-IR spectroscopy, as well as theoretical calculations. Based on the experimental and theoretical phase diagram, we can conclude that the 1:2 TBAB−NA mixture is characterized by a large decrease in the melting point, indicating that it is indeed a deep eutectic mixture and not a simple eutectic mixture. The formation of hydrogen bonds, which is a necessary condition for the formation of a DES, was confirmed by FT-IR spectra. It can be concluded that the hydrogen bond in the DES complexes is formed between the carboxyl group from the NA molecule and the bromine atom from the TBAB molecule. However, only at TBAB:NA molar ratios of 1:1, 1:2, 1:3, 1:4, and 1:5, can it be assumed that hydrogen bonds are formed between the HBA and the HBD. At a molar ratio of 1:10 to 1:30, the spectra are practically indistinguishable from the pure NA spectrum. Therefore, in this case, they are most likely solutions of TBAB in NA, in which hydrogen bonds between acid molecules predominate.

## Supporting Information Available

σ-Profiles of TBA^+^, Br^–^, and NA predicted by COSMO-RS analysis and DSC thermograms of eutectic mixtures and pure DES components (PDF)
