# Supplementary material for: Deep Eutectic Solvents or Eutectic Mixtures? Characterization of Tetrabutylammonium Bromide and Nonanoic Acid Mixtures
Source: J Phys Chem B. 2022 May 24;126(21):3889–96. doi: 10.1021/acs.jpcb.2c00858 (PMC9169048; doi:10.1021/acs.jpcb.2c00858)
Supplement: Supplementary file 1 — jp2c00858_si_001.pdf [file jp2c00858_si_001.pdf]

Supporting information

Deep Eutectic Solvents or Eutectic Mixtures? – Characterization of Tetrabutylammonium Bromide and Nonanoic Acid Mixtures

Andrey Shishov [a], Patrycja Makoś-Chełstowska [b,c*], Andrey Bulatov [a], Vasil Andruch [d]

[a] *Institute of Chemistry, Saint Petersburg State University, RU-198504 Saint Petersburg, Russia*

[b] *Department of Process Engineering and Chemical Technology, Faculty of Chemistry, Gdansk University of Technology, 80–233 Gdańsk, Poland*

[c] *EcoTech Center, Research Centre, Gdańsk University of Technology, G. Narutowicza St. 11/12, 80-233 Gdańsk, Poland*

[d] *Department of Analytical Chemistry, Institute of Chemistry, Faculty of Science, Pavol Jozef Šafárik University in Košice, SK-04154 Košice, Slovak Republic*

*Corresponding author: patrycja.makos@pg.edu.pl

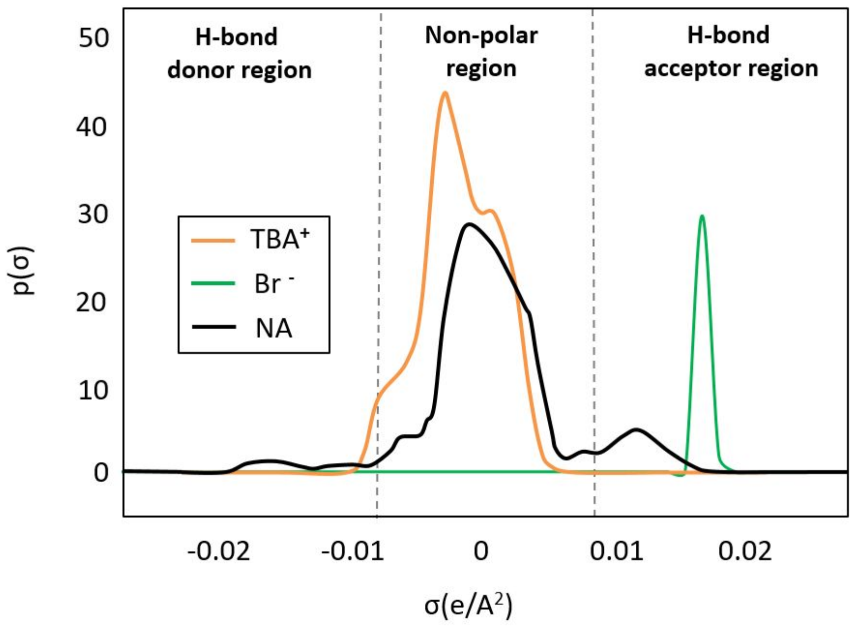

Figure S1 σ-Profiles of TBA^+; Br^-, and NA, predicted by COSMO-RS analysis.

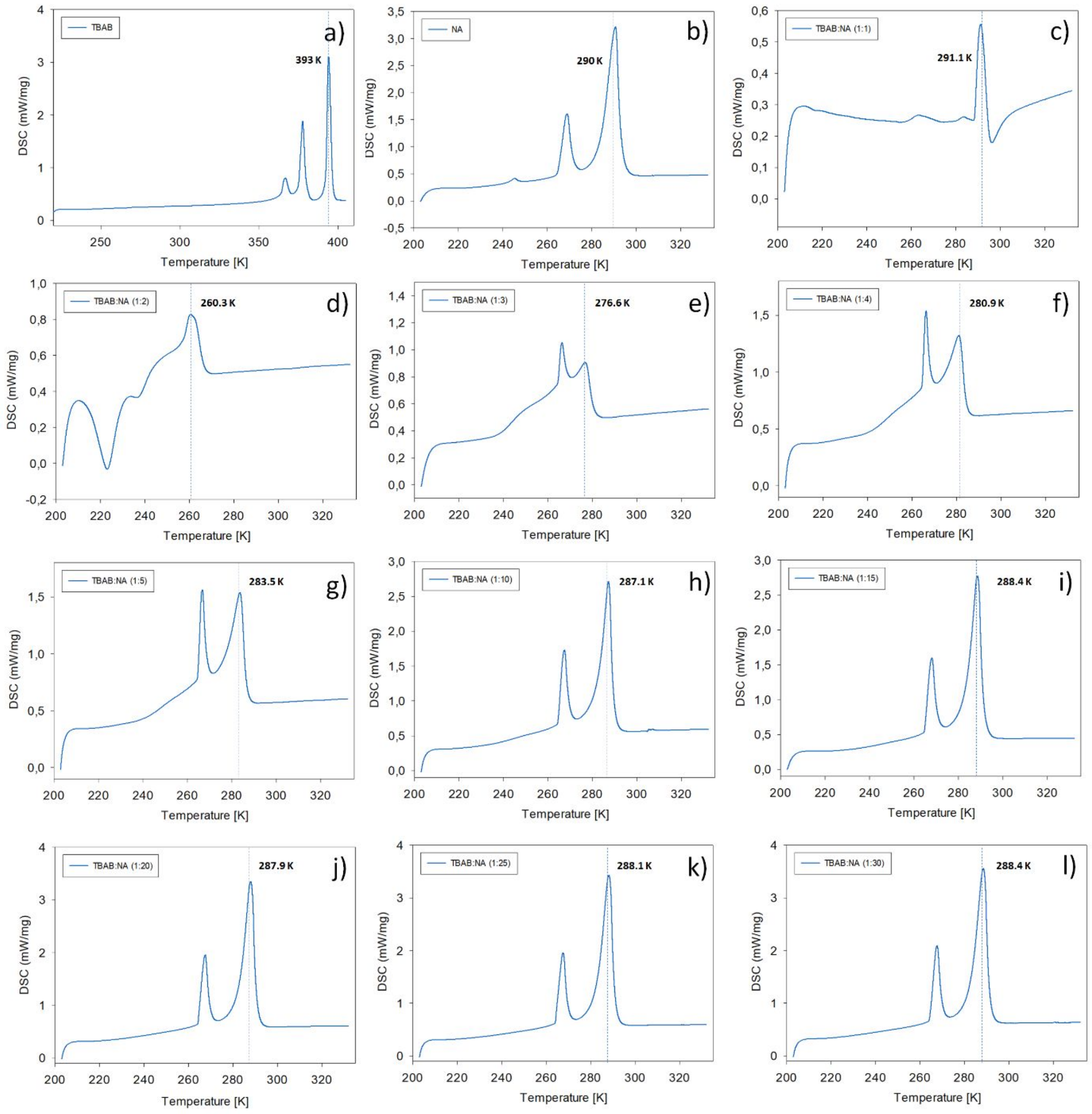

Figure S2 DSC thermograms of a) TBAB; b) NA; c) TBAB:NA (1:1); d) TBAB:NA (1:2); e) TBAB:NA (1:3); f) TBAB:NA (1:4); g) TBAB:NA (1:5); h) TBAB:NA (1:10); i) TBAB:NA (1:15); j) TBAB:NA (1:20); k) TBAB:NA (1:25); l) TBAB:NA (1:30).
